# Atomic‐Scale Mapping of Impurities in Partially Reduced Hollow TiO_2_ Nanowires

**DOI:** 10.1002/anie.201915709

**Published:** 2020-02-07

**Authors:** Joohyun Lim, Se‐Ho Kim, Raquel Aymerich Armengol, Olga Kasian, Pyuck‐Pa Choi, Leigh T. Stephenson, Baptiste Gault, Christina Scheu

**Affiliations:** ^1^ Max-Planck Institut für Eisenforschung GmbH Max-Planck-Straße 1 40237 Düsseldorf Germany; ^2^ Department of Materials Science and Engineering Korea Advanced Institute of Science and Technology (KAIST) 291 Daehak-ro Yuseong-gu Daejeon 34141 Republic of Korea; ^3^ Helmholtz-Zentrum Berlin Helmholtz-Institute Erlangen-Nürnberg 14109 Berlin Germany; ^4^ Department of Materials Royal School of Mines Imperial College London SW7 2AZ UK

**Keywords:** atom probe tomography, electron microscopy, impurities, oxygen vacancies, TiO_2_

## Abstract

The incorporation of impurities during the chemical synthesis of nanomaterials is usually uncontrolled and rarely reported because of the formidable challenge in measuring trace amounts of often light elements with sub‐nanometer spatial resolution. And yet, these foreign elements (introduced by doping, for example) influence functional properties. We demonstrate how the hydrothermal growth and a partial reduction reaction on hollow TiO_2_ nanowires leads to the introduction of parts per millions of boron, sodium, and nitrogen. This doping explains the presence of oxygen vacancies and reduced Ti states at the surface, which enhance the functional properties of TiO_2_. Our results were obtained on model metal oxide nanomaterials and they shed light on a general process that leads to the uncontrolled incorporation of trace impurities in TiO_2_, thereby, having a strong effect on applications in energy‐harvesting.

## Graphical abstract

**Impure thoughts**: Precise characterization of impurities is critical for understanding and enhancing the functional properties of nanomaterials. The structure–property relationships of partially reduced hollow TiO_2_ nanowires are characterized using scanning transmission electron microscopy and atom probe tomography. Oxygen vacancies and undesired boron, sodium, and nitrogen trace impurities are detected on the surface.

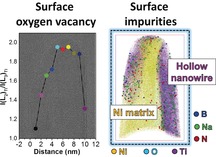

The morphology and porosity of functional nanomaterials determine a variety of properties, such as efficiency, selectivity, and stability of (electro)catalysts and electrodes for energy‐storage applications.^1^ Another critical aspect is that point defects, such as vacancies or foreign elements, can affect the functionality of materials by modifying the band gap, conductivity, catalytic properties, or corrosion behavior.^2^ Besides intentionally added dopants, trace impurities can be introduced within the material during synthesis and post‐treatment procedures. Their detection on a local scale remains a formidable challenge though and tackling it is critical to understanding structure–property relationships and optimizing strategies for the design of nanomaterials.

TiO_2_ is one of the most widely studied metal oxide materials because of its high abundance and superior performance as a photocatalyst, catalyst support, or electrode material for solar cells and metal‐ion batteries.^3^ Stoichiometric TiO_2_ has a band gap of 3.0–3.2 eV, depending on the crystal structure, which only allows absorption of light in the UV region. Partially reduced TiO_2_—also referred as black TiO_2_—has been widely studied because of its visible‐light absorption, which is enabled by a small band gap and excellent metal‐like conductivity.^4^ These beneficial properties are generally attributed to the surface disorder associated with the presences of oxygen vacancies and titanium Ti^3+^ species, as well as other non‐stoichiometric regions within the crystal that induce band gap states.^5^ Controlling the presence of the metastable, defective, and off‐stoichiometric TiO_2_ at the surface of nanostructured morphologies is critical to achieve enhanced properties.^6^

Sodium borohydride (NaBH_4_) can be used as a reductant under an inert gas atmosphere to produce black TiO_2_,3a which has the advantage of higher safety compared to use of hydrogen gas for reduction.^7^ Additionally, employing a lower reaction temperature during the reduction process when NaBH_4_ is used renders this method applicable to TiO_2_ grown on temperature‐sensitive transparent glass electrodes, such as fluorine‐doped tin oxide (FTO). TiO_2_/FTO assemblies are typically used for (photo)electrochemical applications, and they would lose their good conductivity if exposed to high temperature.^8^ However, whether trace elements from the inert gas (such as N_2_), or impurities from the reductant (sodium and boron) are introduced and their possible effect on the structure and properties of TiO_2_, are still unclear.^9^ These elements can introduce disorder in the TiO_2_ crystal lattice and can lead to a lower band gap. N can act as an anionic dopant of TiO_2_ since its atomic p‐orbital levels are appropriate for narrowing the otherwise wide band gap of TiO_2_.^10^ B in TiO_2_ can have a dual effect by narrowing the band gap and compensating for the excess charge from N doping.^10^, ^11^ It is suggested that interstitial B stabilize Ti^3+^ species.^12^ The role of Na in TiO_2_ remains unclear, although Na can act as a recombination center or limit the crystallization of TiO_2_.^13^

Herein, we studied partially reduced hollow TiO_2_ nanowires (R‐HTNWs) using (scanning) transmission electron microscopy ((S)TEM) and atom probe tomography (APT) to demonstrate the feasibility of our approach. The 3D morphology of the R‐HTNWs was characterized using electron tomography, while the local distribution of oxygen vacancies—more specifically the correlated Ti^3+^ species—was analyzed using electron energy loss spectroscopy (EELS). To enable APT analysis, electrodeposition was applied to embed R‐HTNWs in a Ni matrix, which also fills the hollow core of the NW. The 3D hollow structure of R‐HTNWs was successfully reconstructed and the distribution of small amounts of B, Na, and N resulting from the hydrothermal growth and subsequent reduction with NaBH_4_ under N_2_ atmosphere were quantified. Our results characterize the 3D structure and impurities of porous metal oxide nanomaterials at an atomic scale and reveal that trace impurities are located mostly at the surface region of the NWs. This information is necessary for determining structure–property relationships.

Hollow TiO_2_ nanowires (HTNWs) were grown on a FTO glass substrate using a hydrothermal method and subsequently reduced to R‐HTNWs using NaBH_4_ under N_2_ flow (Supporting Information). Scanning electron microscopy (SEM) and TEM images show the hollow morphology of the R‐HTNWs, which are composed of an assembly of nanofingers (Figures 1 a,b). This is confirmed by the side‐view TEM image in Figure 1 c. Lattice distances of 2.9 and 3.2 Å are obtained from one R‐HTNWs nanofinger, which are assigned to rutile (001) and (110), respectively (Figure 1 d). The contrast difference in negative spherical aberration imaging in an aberration‐corrected TEM allows us to resolve the atomic columns containing Ti−O or only Ti when viewed along the rutile [1$\bar{1}$ 0] direction (Figure 1 e; Supporting Information, Figure S1).

**Figure 1:**
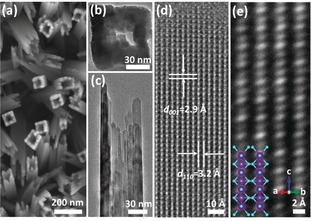
a) SEM and b–e) TEM images of R‐HTNWs. e) Atomic resolution image with the projected crystal structure of rutile TiO_2_ viewed in the [1$\bar{1}$ 0] direction; Ti (purple), O (cyan).

The chemistry of R‐HTNWs was obtained from energy‐dispersive X‐ray spectroscopy (EDS) and EELS in STEM mode. The side‐view high‐angle annular dark‐field (HAADF)‐STEM image and EDS elemental maps of Ti and O in Figure 2 a display the hollow morphology with a homogeneous distribution of both Ti and O. Similar information is obtained in top‐view and in EELS elemental maps (Figure S2). In accordance with the literature,^5^ the surface region of the nanowire and the individual nanofingers have a larger number of Ti^3+^ species compared to the core (Figures 2 b,c). This information is obtained by analyzing the EELS Ti L_2,3_‐ and O K‐edges. The Ti L_2,3_‐edge in the core of the nanofingers has four peaks resulting from the spin‐orbit splitting into Ti L_3_ (2p_3/2_) and Ti L_2_ (2p_1/2_) edges with further crystal field splitting of Ti 3d states into t_2g_ and e_g_ (Figure 2 c). The four peaks observed here are characteristic for crystalline TiO_2_ phases such as anatase and rutile.^14^ The left shoulder on the e_g_ peak of Ti L_3_ is typical for the rutile nature6b and confirms the high‐resolution TEM images, which indicate that the interior has a rutile structure. The EELS Ti L_2,3_‐edge of the surface regions do not show the typical split into four peaks and they occur at a slightly lower energy loss, which indicates the presence of Ti^3+^ species. Spectra are obtained from one surface though the middle to the other surface with approximate 1 nm steps (Figure 2 b). A high I(L_2_)_Ti_ to I(L_3_)_Ti_ indicates a high Ti^4+^ concentration. The value in the core part of a single nanofinger decreases toward both surfaces, revealing a surface‐specific introduction of oxygen vacancies and their gradient distribution. This result demonstrates that oxygen vacancies, or other impurities stabilizing Ti^3+^, are concentrated at the surface and the distorted rutile structure region.

**Figure 2:**
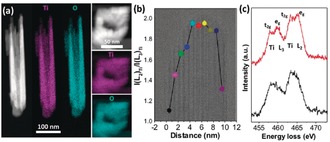
a) HAADF‐STEM images and STEM‐EDS maps for both side‐ and top‐views of R‐HTNWs. b) A TEM image of a R‐HTNW nanofinger and overlaid I(L_2_)_Ti_/I(L_3_)_Ti_ values determined from the EELS Ti L_2,3_ data at each position. The I(L_2_)_Ti_/I(L_3_)_Ti_ ratio was obtained by averaging over a box with a size of ca. 1×10 nm^2^ with the long axis parallel to the wire surface. c) The Ti L_2,3_‐edge for the surface region (black spectrum) and ca. 6 nm away (red spectrum). Note: the black spectrum contains contributions from both surface and bulk atoms.

To understand the 3D morphology of an entire R‐HTNWs, electron tomography was performed based on HAADF‐STEM images. The data were also used to determine the regions of the APT reconstruction where only a part of the wire is analyzed. The HAADF‐STEM images from R‐HTNWs tilted in the range of ±60° were reconstructed to obtain a 3D volume. Figures 3 a–c summarize representative HAADF‐STEM images taken at 0°, −60°, and +60° tilt angles, demonstrating changing contrast by tilting the sample. The 3D reconstructed volume shows the nanofingers as well as the empty space inside the nanowire (Figure 3 d; Supporting Information, Movie 1). The cross‐sectional cut through the reconstructed 3D volume presents the hollow nature of R‐HTNWs in detail (Figure 3 e).

**Figure 3:**
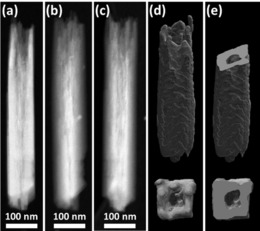
Representative HAADF‐STEM images of a R‐HTNW from a) 0, b) −60, and c)+60 ° tilt angles. d) Reconstructed volume and e) sections of the R‐HTNW.

To quantify possible trace impurities, such as B, Na, and N, that are known to change the local crystal structure and properties of TiO_2_, we used APT. The low electrical conductivity of TiO_2_ and the presences of pores in the R‐HTNWs were obstacles for conventional APT characterization. Filling pores with a highly conductive material has been shown to be a solution for stable and homogeneous electrostatic field distribution,^15^ but has not been demonstrated for poorly conducting, porous nanomaterials. Herein, we co‐electrodeposited R‐HTNWs, which detached from the FTO along with Ni to form a film on a Cu substrate, and allowed preparation of an APT specimen using focused ion beam (FIB) milling.^16^ TiO_2_ has been reported as inert particles during a co‐electrodeposition process of Ni films.^17^ The electrodeposition depends strongly on the surface heterogeneity.^18^ We observed protrusions on the co‐electrodeposited Ni surface, which was indicative of the presence of the embedded nanowires (Figure S3). The nanowires inside the protrusion were confirmed by cross‐sectioning one of these regions with the FIB (Figure S4a). The hollow morphology of the R‐HTNWs were preserved after co‐electrodeposition with Ni. The pores in the R‐HTNWs were successfully filled with Ni without any detectable voids, which could induce inhomogeneous field evaporation during APT measurements. Every protrusion we investigated contained R‐HTNWs, helping us to guide the fabrication of a site‐specific APT specimen. One of these regions was then lifted out, placed onto a Si microtip, sliced to only contain the R‐HTNWs, and sharpened into a needle‐like specimen (Figures S4b,c).

Details of the APT analysis of the R‐HTNWs embedded in Ni can be seen in Figure S5. The major peaks in the mass‐to‐charge ratio spectrum are assigned to Ti−O molecular ions and the electrodeposited Ni matrix in single‐ and double‐charged states (Table S1). A strong O^+^ peak originating from the R‐HTNWs is detected at 16 Dalton (Da). Several peaks are clearly assigned to atomic species introduced during the reduction of R‐HTNWs. The peaks at 14 and 23 Da are N^+^ and Na^+^ ions, respectively, and the peaks at 10 and 11 Da correspond to B^+^ ions. The origin of the impurities is discussed in the proceeding text.

A 3D atom map containing a part of the R‐HTNW embedded in Ni was reconstructed for the hollow nanowire (Figure 4 a; Movie 2) and for the single nanofinger regions (Figure 4 b). Reconstructed Ti and O atoms, and an iso‐composition surface of 15 at % Ti, are presented within the Ni matrix. The 20 and 10 nm thick slices for the reconstructed hollow nanowire region and a single nanofinger region, respectively, show the distribution of other elements such as B, Na, and N when viewed along the *y*‐axis. These elements are concentrated along the surface region in accordance with oxygen vacancies detected with EELS.

**Figure 4:**
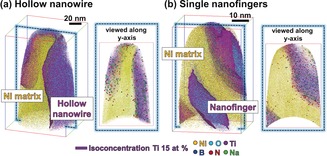
3D atom maps of a) hollow nanowires and b) single nanofingers of R‐HTNWs embedded within Ni.

The distribution of B, Na, and N atoms in the R‐HTNWs is quantified by a composition profile calculated as a function of the distance to the isosurface (proximity histograms) defined above and plotted in Figure 5 a. The concentration of Ti and O reach 34 and 63 at %, respectively, in the bulk of the nanowire region. The ratio of Ti to O concentration is roughly 1.9, which is close to the stoichiometry of TiO_2_. B, Na, and N are detected inside this bulk region too, which indicates that these elements are incorporated during the hydrothermal growth and reduction procedure. In this part of the nanowire, the atomic concentrations of B, Na, and N are 0.31±0.02, 0.22±0.01, and 0.026±0.004 at %, respectively. The surface of the R‐HTNW, marked by the intersection between the Ni matrix and Ti−O, sees a strong enrichment in B, Na, and N. A proximity histogram from a single nanofinger region in R‐HTNW shows similar results compared to the hollow nanowire region (Figure 5 b).

**Figure 5:**
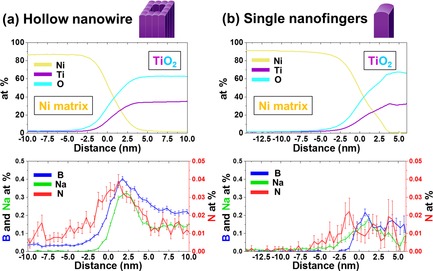
Proxigram concentration profiles of a) hollow nanowires and the b) single nanofinger regions in R‐HTNWs. Top: Ni, Ti, and O. Bottom: B, Na, and N; 15 at % Ti.

The interfacial excess value (*Γ_i_*) of B, Na, and N from both hollow nanowire and single nanofinger regions can be determined from the impurity concentration detected at the surface (for calculations see the Supporting Information). The values of *Γ_i_* after background correction, for both the hollow nanowire and single nanofinger, are presented in Table S2. They differ by a factor of about 2. This is because, in the calculation, all four {110} surface planes were considered as the exposed surface area, two of which are actually blocked by other nanofingers (Figure S6).

To address the origin of the impurities, an APT analysis was conducted on the non‐reduced HTNWs. Figure S7 shows the atom map and element composition profile of such a HTNW. B is not detected here (Table S3), confirming that the source of B impurity on the surface of R‐HTNWs is the NaBH_4_ used for the reduction step. A low amount of Na (0.21±0.02 at %) is detected in the HTNWs, indicating that the presences of Na in R‐HTNWs can originate from the NaBH_4_ used for the reduction as well as from the diffusion of Na from the glass‐based FTO substrate during the hydrothermal growth of the HTNWs.^13^ The low amount of N (0.013±0.002 at %) detected in the non‐reduced state can originate from adsorption during specimen transfer or their processing. The higher amount of N (0.026±0.004 at %) in the R‐HTNWs can be attributed to higher surface oxygen vacancies, which represent favorable sites for the adsorption of N_2_.^19^ The result reveals that impurities of Na, B, and N have a strong tendency to be deposited on the exposed {110} surface of the R‐HTNWs.

The surfaces need to be considered when discussing structure–property relationships of R‐HTNWs as their impact can be very large.^20^ First, the unintentionally added surface impurities contribute to distorting the rutile crystal structure. This distortion is responsible for higher carrier mobility, leading to metallic conductivity.^21^ The surface impurities also stabilize Ti^3+^ on the surface as interstitial B allows sufficient Ti^3+^ species in TiO_2_.^12^, ^22^ This is supported by our STEM‐EELS measurements. B and N synergistically reduce the band gap of TiO_2_, increasing the performance for photo(electro)catalysis and energy‐storage applications.^11^ Additionally, surface B increases electrocatalytic properties in fuel cell applications as a consequence of its high oxygen adsorption ability.^22^ Our preliminary electrochemical data shows that the properties of these NWs are governed by the presences of vacancies and associated Ti^3+^ states, and their performance improves with increasing vacancy concentration (Figure S8). This reinforces the importance of measuring low levels of impurities, since they underpin the formation and stabilization of oxygen vacancies and the more active Ti^3+^ states. Our approach blends (S)TEM, EELS, and APT sheds light on near‐atomic‐scale structure–property relationships, which help to drive the optimization of functional nanomaterials.

In summary, our work shows that the detection of trace elements enabled by the combination of (S)TEM and APT is necessary to further understand the functional properties of TiO_2_, but will also be suitable for other nanomaterials. The lack of precise, quantitative characterization of impurities likely explains contradicting results reported in the literature, since different amounts of trace elements might be introduced during the synthesis and were, up to now, most often not considered. The approach discussed herein could readily be deployed to a vast array of different material systems and hence lead to better control over the integration of impurities with an aim to enhance the materials’ functional properties.

## Conflict of interest

The authors declare no conflict of interest.

## Supporting information

As a service to our authors and readers, this journal provides supporting information supplied by the authors. Such materials are peer reviewed and may be re‐organized for online delivery, but are not copy‐edited or typeset. Technical support issues arising from supporting information (other than missing files) should be addressed to the authors.

Supplementary

Supplementary

Supplementary
