# Supplementary material for: Atomic‐Scale Mapping of Impurities in Partially Reduced Hollow TiO2 Nanowires
Source: Angew Chem Int Ed Engl. 2020 Feb 7;59(14):5651–5. doi: 10.1002/anie.201915709 (PMC7155045; doi:10.1002/anie.201915709)
Supplement: Supplementary file 1 — Supplementary [file ANIE-59-5651-s001.pdf]

Angewandte Chemie
Eine Zeitschrift der Gesellschaft Deutscher Chemiker

Supporting Information

Atomic-Scale Mapping of Impurities in Partially Reduced Hollow TiO_2 Nanowires

Joohyun Lim[+], Se-Ho Kim[+], Raquel Aymerich Armengol, Olga Kasian, Pyuck-Pa Choi, Leigh T. Stephenson, Baptiste Gault,* and Christina Scheu**

anie_201915709_sm_miscellaneous_information.pdf
anie_201915709_sm_Movie1.avi
anie_201915709_sm_Movie2.mp4

Supporting Information

Experimental Procedures

Synthesis of R-HTNWs

Rutile TiO_2 nanowires were grown on a FTO glass substrate using a modified hydrothermal method.[1] They are composed of finer nanofinger bundles surrounded by compact and bigger outer nanofingers (Figure S9). HTNWs were obtained by selective core etching of TiO_2 nanowires with a 1:1 (v/v) mixture solution of deionized water and hydrochloric acid (HCl, 36 wt%, Sigma Aldrich) in an autoclave reactor at 180ºC for 2h.[2] Finally, R-HTNWs were prepared by the reduction of HTNWs with $NaBH_4$ (Sigma Aldrich) under N_2 flow at 400 ºC for 2h with a heating rate of 10ºC/min. The obtained R-HTNWs were washed using deionized water and dried at room temperature.

Co-deposition of R-HTNWs within an electrodeposited Ni

R-HTNWs were encapsulated within a Ni film according to the modified procedure developed by Kim et al.[3] For Ni electrolyte preparation, 15 g of nickel sulfate hexahydrate ($NiSO_4 \cdot 6H_2O$, Sigma Aldrich) and 2 g of boric acid (H_3BO_3, Sigma Aldrich) were dissolved in 50 mL of distilled water (18.2 MΩ-cm) from the AQUA Solution type 1. Then, the R-HTNWs were dispersed in the Ni electrolyte using a sonicator for 10min and the mixed solution was poured into a vertical cell for co-electrodeposition process. Co-deposition was carried out in a specially designed vertical cell including a Cu substrate and a Pt-mesh counter electrode as shown in Figure S10a and b. In order to homogenously deposit Ni on the Cu substrate and effectively deposit the nanowires within the Ni layer, a vertical cell is designed in a conical shape. The surface area of Pt counter electrode (2 cm^2) is larger than that of the Cu working electrode (0.2 cm^2). The one-step co-electrodeposition was performed at a constant current of 19 mA for 500s. In Figure S10c, a photograph of the co-electroplated Ni and R-HTNWs on a Cu substrate is shown.

Electron microscopy characterization

SEM was performed to investigate the morphology of the R-HTNWs (Gemini 500, Zeiss, in-lens detector, 2 kV). (S)TEM was performed using a FEI 60-300 Titan Themis operated at 300 kV with a Cs-corrector for the probe forming lens. A rutile TiO_2 model was applied to simulate a TEM image using the QSTEM image simulation software.[4] We used the same optical parameters as the values used experimentally. The chemical composition of R-HTNWs was analyzed by EDS in the STEM mode. EELS data were acquired in the STEM mode using a dual channel acquisition mode[5] with a dispersion of 0.05 eV per channel and a pixel time of 2-3s using a Gatan software. All spectra were corrected for channel to channel gain variations and dark current.[6] The background was subtracted using a standard power law. Post-edge background of Ti–$L_{2,3}$ edge was further removed with a double arctangent step function. To differentiate the effect of the reducing agent and the electron beam bombardment during the EELS measurements on the reduction of the TiO_2, EELS data were acquired for the R-HTNWs and HTNW under similar illumination conditions (Figure S11a).

X-ray photoelectron spectroscopy (XPS)

XPS was used to analyze the oxidation state of TiO_2 before and after reduction step (Figure S11b). XPS was carried out using a Quantera II (Physical Electronics, Chanhassen, MN, USA) equipped a monochromatic Al Kα X-ray source (1486.6 eV) operated at 15 kV and 25 W. The C 1s signal at 285.0 eV was used as a reference for the binding energy scale. The experimentally obtained spectra was analyzed using the Casa XPS software.

Electron tomography

HAADF-STEM images were acquired from the R-HTNW every 5º tilt angle over ±60º tilting range for the 3D reconstruction using a JEOL JEM-2200FS TEM at 200 kV. The obtained STEM images were aligned using the TomoJ plugin installed in ImageJ. Simultaneous iterative reconstruction (SIRT)[7] and discrete algebraic reconstruction technique (DART)[8] were applied to reconstruct the final 3D volume of the R-HTNW.

APT characterization

A needle-shape specimen was prepared from the co-deposited sample using FIB (Helios NANOLAB 600i, FEI) for APT measurement to identify the chemical composition and position for each element.[9] APT analyses were performed using a local electrode atom probe (Cameca LEAP 5000 XS system) in pulsed UV laser mode at a detection rate of 1 %, a laser pulse energy of 80 pJ, and a pulse frequency of 125 kHz. The specimen temperature was set to ~50K during analysis. The set parameter was based on the recent

report on natural rutile TiO_2 measurement for atom probe tomography.[10] Data reconstruction and analyses were performed using the commercial software Imago visualization and analysis system standard (IVAS) 3.8.2 developed by Cameca Instruments. All 3D atom maps presented in this paper were reconstructed using the standard voltage reconstruction protocol.

Results and Discussion

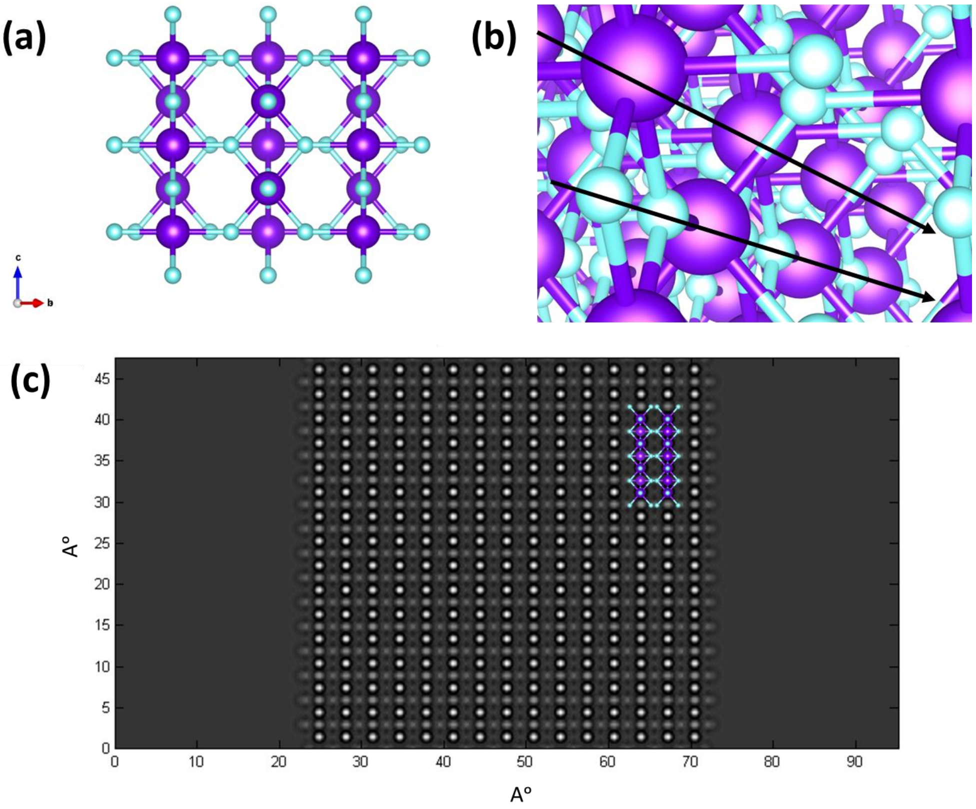

Figure S1. A model and a simulated TEM image of rutile TiO_2. (a) Parallel- and (b) slightly tilted perspective views of $[1\bar{1}0]$ direction. (c) A simulated TEM image super-imposed with a rutile TiO_2 model in $[1\bar{1}0]$ direction. Purple and cyan spheres represent Ti and O atoms, respectively.

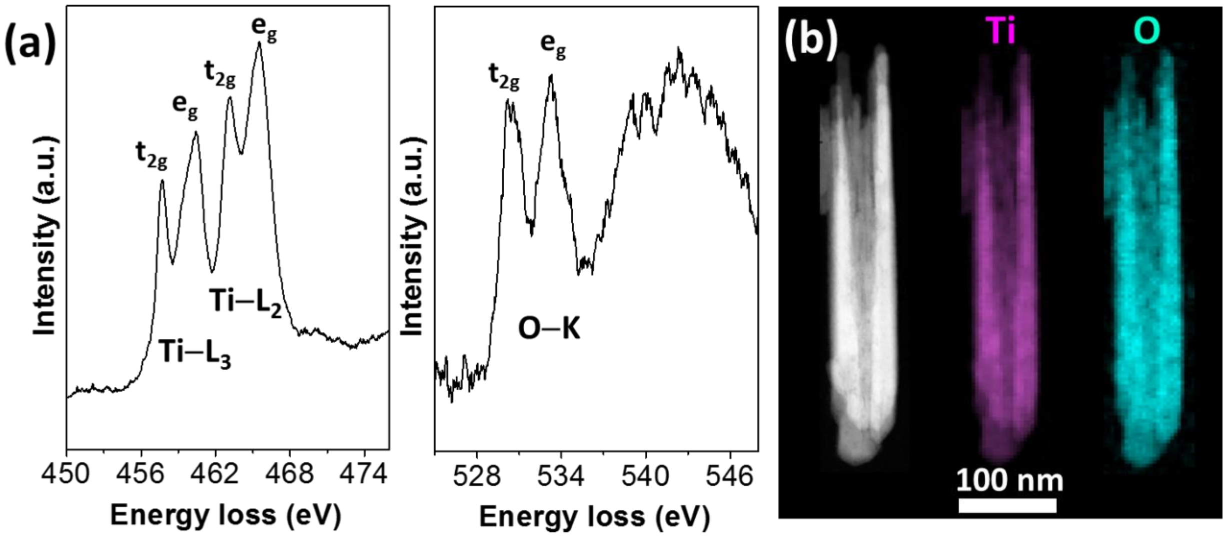

Figure S2. (a) STEM-EELS Ti–$L_{2,3}$ and O–K edges of an entire R-HTNW. (b) ADF-STEM image and STEM-EELS maps of R-HTNW.

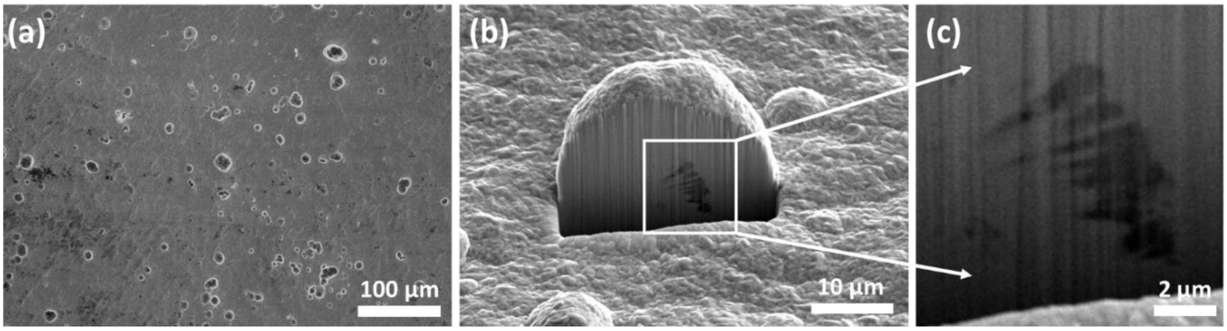

Figure S3. SEM images of (a) protrusions on the surface of Ni co-deposited with R-HTNWs and (b) and (c) cross-section of a protrusion.

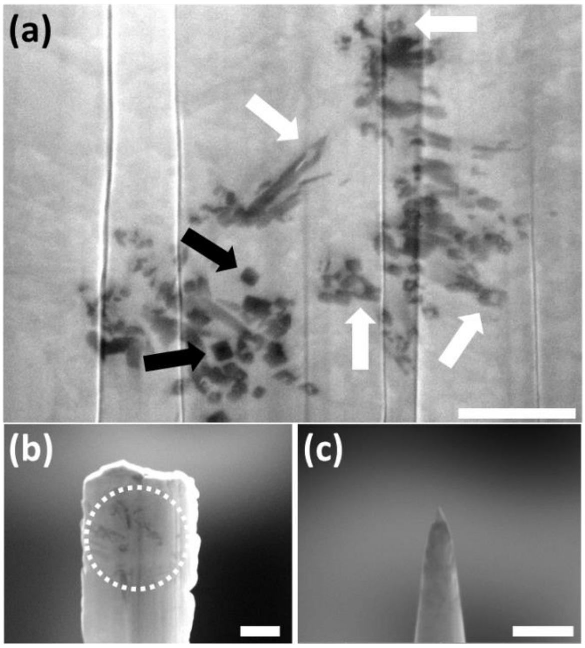

Figure S4. (a) Cross-sectional SEM image of R-HTNWs embedded in Ni. SEM images of the cut and lifted out area (b) before and (c) after final sharpening process. White scale bars indicate 500 nm.

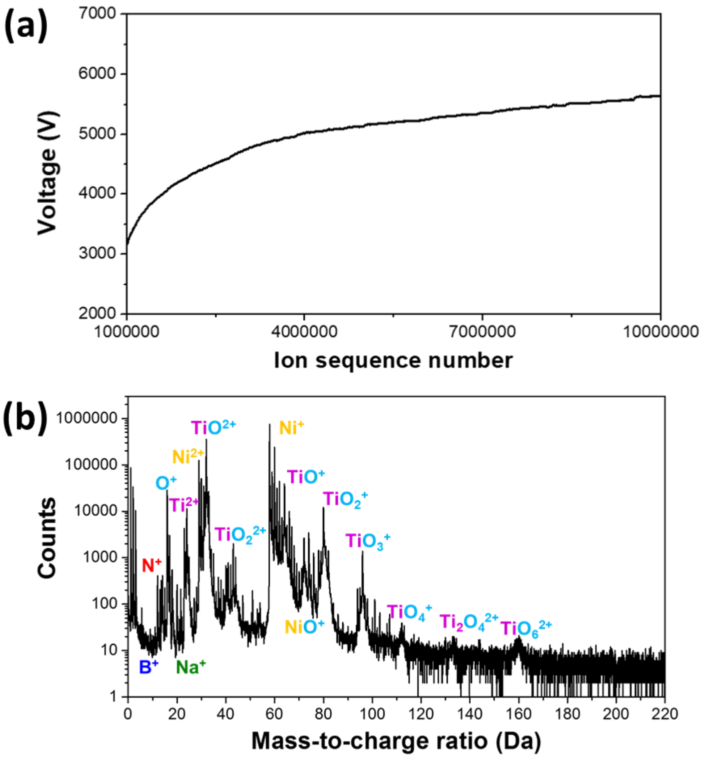

Figure S5. a) Voltage history curve for R-HTNW in Ni matrix during evaporation. The steady increase in voltage over the course of the APT measurement indicates a high reliability of our specimen preparation approach and the complete filling of the empty space in R-HTNW. b) Overall mass spectrum of R-HTNW embedded in Ni matrix.

Table S1. List of analyzed peak numbers from R-HTNW embedded in Ni matrix.

Chemical species	Charge state	Mass-to-charge ratio, m/z (Da)
Ni	1+	58, 60, 61, 62, 64
	2+	29, 30, 30.5, 31, 32
NiH	1+	59, 61, 62, 63, 65
NiO	1+	74, 75, 76, 77, 78, 79, 80, 81
	2+	37, 37.5, 38, 38.5, 39, 39.5, 40, 40.5
Ti	1+	56, 57, 58, 59, 60
	2+	23, 23.5, 24, 24.5, 25
TiO	1+	62, 63, 64, 65, 66, 67
	2+	31, 31.5, 32, 32.5, 33, 33.5
TiO_2	1+	78, 79, 80, 81, 82, 83, 84
	2+	39, 39.5, 40, 40.5, 41, 41.5, 42
TiO_3	1+	94, 95, 96, 97, 98, 99, 100, 101
	2+	47, 47.5, 48, 48.5, 49, 49.5, 50, 50.5

Supplementary Text

Surface density of impurities

The Gibbsian interfacial excess (Γ_i) of minor elements such as B, Na, and N atoms at the R-HTNW/Ni matrix interface are calculated using:[11]

$$\Gamma_i = \frac{1}{A\mu} N_i^{excess} = \frac{1}{A\mu}\left(N_i^{total} - N_i^{Ti} - N_i^{Ni}\right)$$

Here *A* is the surface area from the R-HTNW and *μ* is the detection efficiency (~80%) of the LEAP 5000 XS.[12] The excess number of impurity element *i* associated with the TiO_2/Ni interface (N_i^{excess}) is determined by comparing total number of *i* element (N_i^{total}) to a reference system where the number of element *i* in R-HTNW (N_i^{Ti}) and Ni matrix (N_i^{Ni}) is used.

Table S2. Surface density values for B, Na, and N for hollow nanowire and single nanofinger.

Γ_i (atom/nm^2)	Hollow nanowire (R-HTNW)	Single nanofingers (R-HTNW)	Ratio
B	0.87	0.52	1.7
Na	0.49	0.26	1.9
N	0.086	0.047	1.8

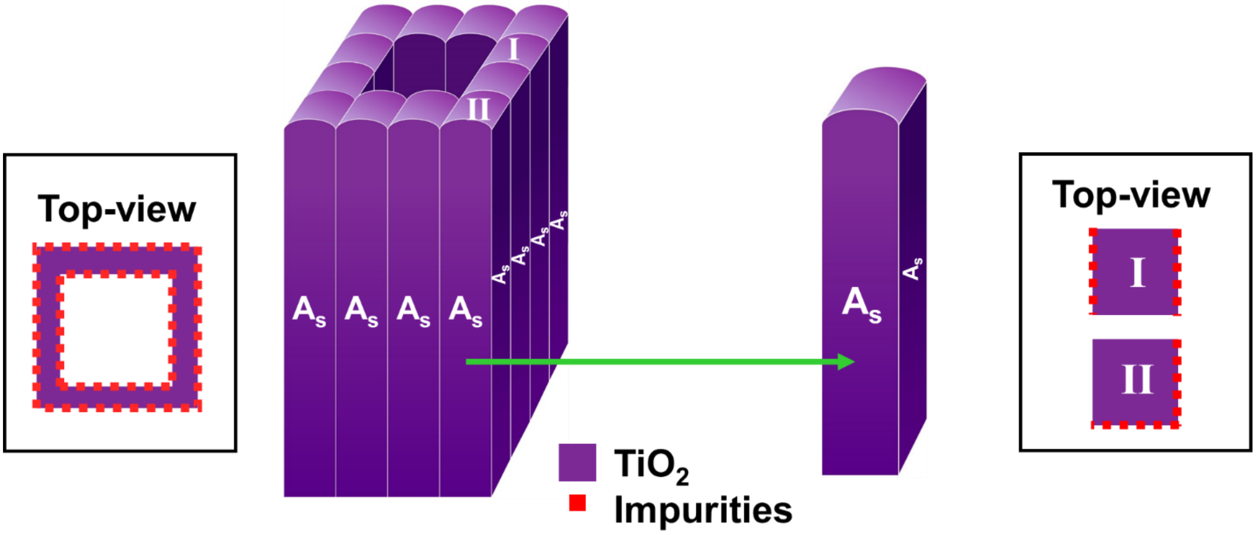

Figure S6. Schematic image for exposed surface of hollow nanowire and single nanofinger where impurities are mainly present. Inset image of top-view of single nanofingers surrounded by others (I) or at the corner (II) with possible impurities distribution.

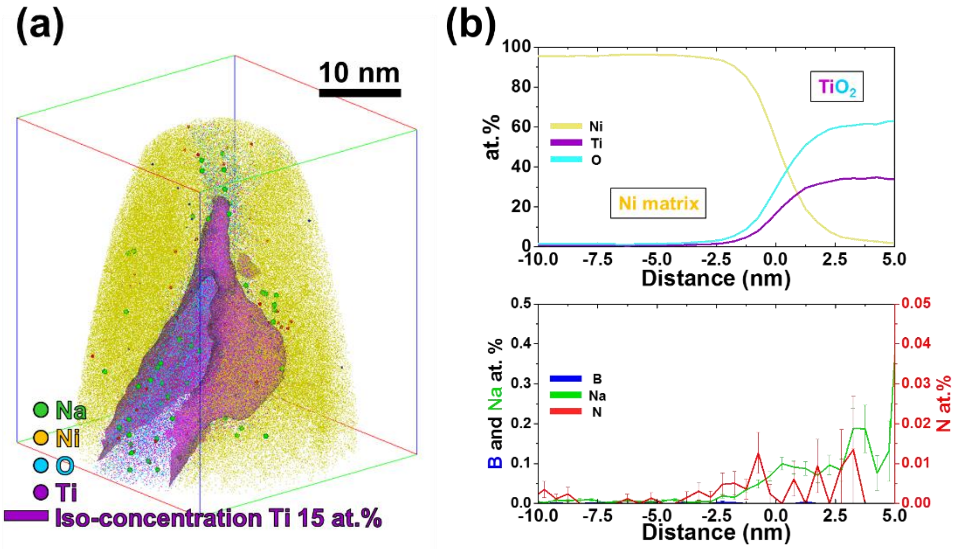

Figure S7. (a) 3D atom map of a HTNW embedded within Ni using an iso-concentration surface of Ti at 15 at.% (purple). (b) Proxigram concentration profile of major (Ni, Ti, and O) and minor (B, Na, and N) elements along iso-concentration of 15 at.% Ti. Yellow, cyan, purple, blue, green, and red dots represent the reconstructed atomic position of Ni, O, Ti, B, Na, and N, respectively.

Table S3. Bulk composition for for B, Na, and N in R-HTNW and HTNW.

Elements (at.%)	Hollow nanowire (R-HTNW)	Single nanofingers (R-HTNW)	HTNW
B	0.31±0.02	0.35±0.02	-
Na	0.22 ±0.01	0.19 ±0.02	0.21±0.02
N	0.026±0.004	0.028 ±0.007	0.013±0.002

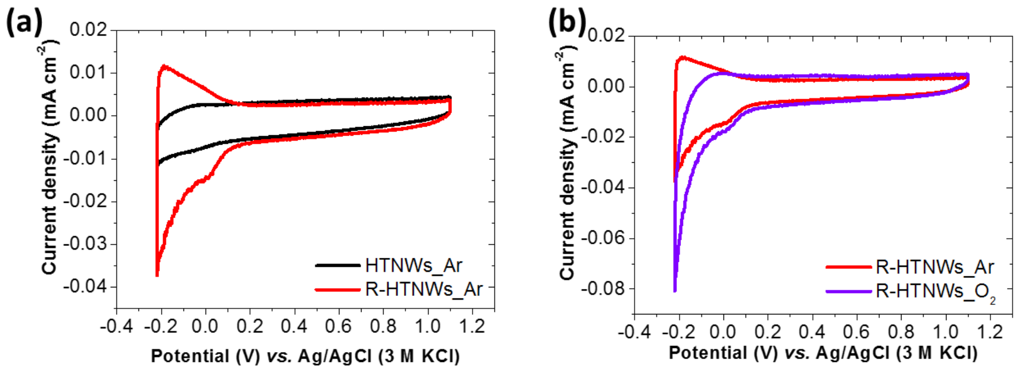

Figure S8. Cyclic voltammetry curves of (a) HTNWs and R-HTNWs under Ar and (b) R-HTNWs under Ar or O_2 monitored with 0.2 V/s in 0.1 M $HClO_4$.

Preliminary cyclic voltammetry (CV) data reveals a different electrochemical behavior of R-HTNWs compared to HTNWs (Figure S8a). Specifically, the CV curves of the R-HTNWs have two characteristics, which are not observed for the HTNWs. Firstly, a small reduction in the signal is found at ~0 V vs. Ag/AgCl/3M KCl under Ar flow. This can be assigned to proton assisted reduction of Ti^{4+} to Ti^{3+} in the presence of dopant atoms such as boron.[13] Secondly, R-HTNWs show a larger current density between about -0.2 to 0.1 V compared to HTNWs, which can be attributed to an increase in the proton insertion/extraction into the rutile lattice enabled by the higher vacancies concentration.[14] Proton insertion/extraction is a key process in metal-ion batteries and electrochromic batteries and displays.[15] The CV of R-HTNWs exhibits a higher current density under O_2 atmosphere compared to under Ar atmosphere (Figure S8b). This is typical for materials with a high reactivity for oxygen reduction reaction which is important for metal-air batteries and fuel cell applications.[16]

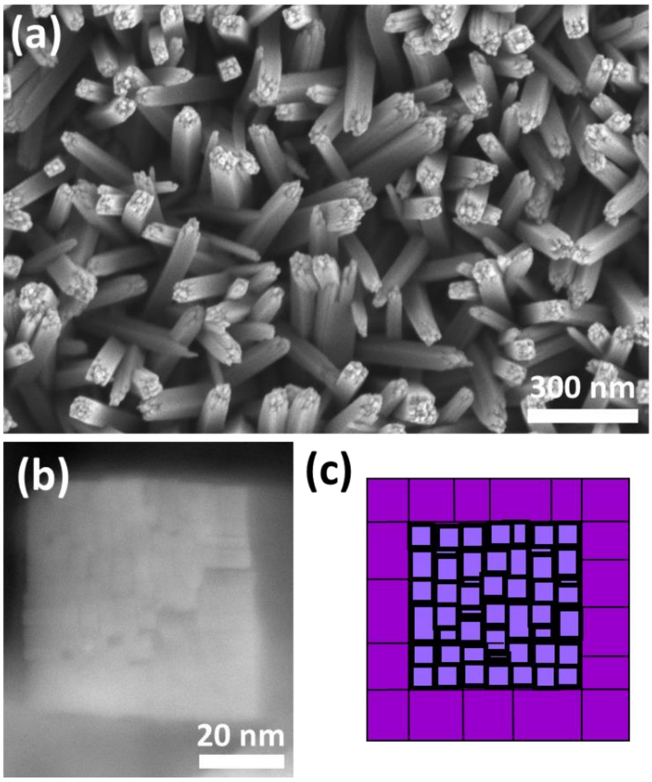

Figure S9. Top-view of (a) SEM, (b) STEM, and (c) schematic images of TiO_2 nanowires.

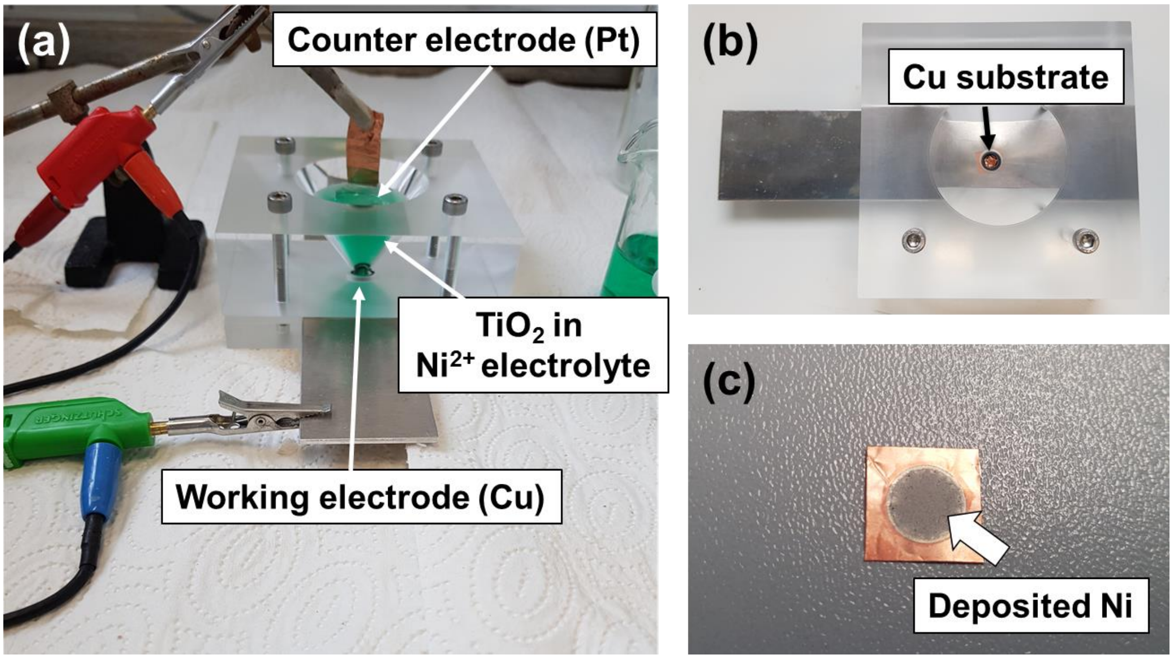

Figure S10. Photographs of the one-step co-deposition set-up. (a) A designed vertical cell with a Pt counter electrode (top) and a Cu working electrode (bottom). The TiO_2 dispersed Ni electrolyte is poured into the cell for co-deposition process. (b) Top view of the designed cell. (c) An example of the co-deposited sample of the R-HTNWs and Ni film.

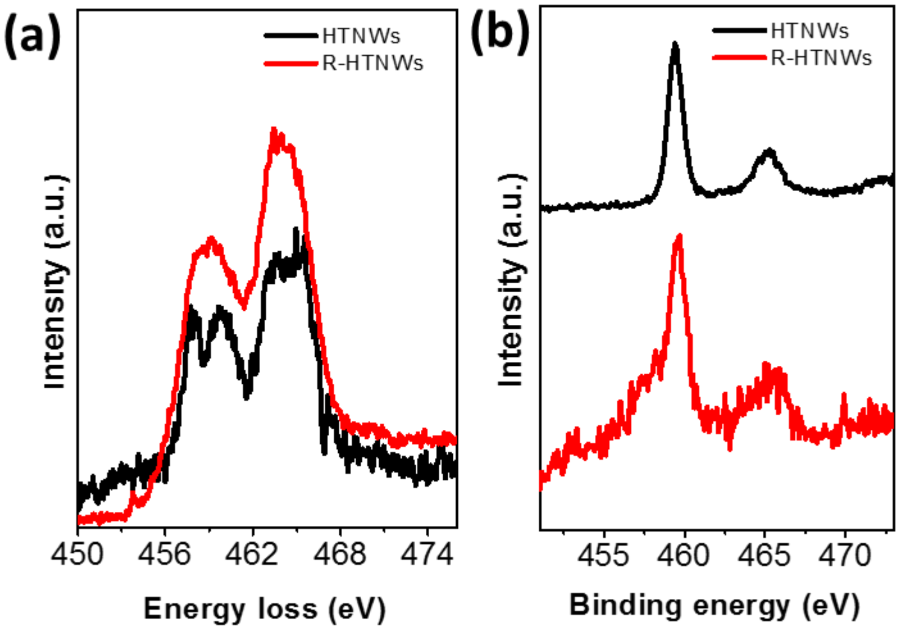

Figure S11. (a) EELS Ti–$L_{2,3}$ edge acquired for a single nanofinger and (b) Ti 2p XPS spectra of R-HTNWs and HTNWs.

No splitting is observed for the Ti–L_2 and Ti–L_3 peaks in the R-HTNW single nanofingers and the peaks are occurring at slightly lower energy loss when compared to that of HTNWs single nanofingers (Figure S11a). This indicates the presence of oxygen vacancies with the partial reduction of Ti^{4+} to Ti^{3+}, inducing local distortions of the rutile structure. Although TiO_2 in our TEM experiments could be slightly reduced by the electron beam bombardment, EELS indicates that difference in the nanowire reduction step is the main origin of the changes in the oxidation state of Ti. Ti 2p XPS spectra also show an additional shoulder peak ~457 eV only in R-HTNWs, indicating the presence of Ti^{3+} after the reduction reaction (Figure S11b).

References

[1] a) B. Liu, E. S. Aydil, *J. Am. Chem. Soc.* **2009**, *131*, 3985-3990; b) A. Folger, P. Ebbinghaus, A. Erbe, C. Scheu, *ACS Appl. Mater. Interfaces* **2017**, *9*, 13471-13479.
[2] L. Pan, H. Huang, C. K. Lim, Q. Y. Hong, M. S. Tse, O. K. Tan, *RSC Adv.* **2013**, *3*, 3566-3571.
[3] S.-H. Kim, P. W. Kang, O. O. Park, J.-B. Seol, J.-P. Ahn, J. Y. Lee, P.-P. Choi, *Ultramicroscopy* **2018**, *190*, 30-38.
[4] C.Koch, Determination of Core Structure Periodicity and Point Defect Density Along Dislocations, Arizona State University, 2002, Dissertation.
[5] J. Scott, P. J. Thomas, M. MacKenzie, S. McFadzean, J. Wilbrink, A. J. Craven, W. A. P. Nicholson, *Ultramicroscopy* **2008**, *108*, 1586-1594.
[6] R. F. Egerton, *Rep. Prog. Phys.* **2008**, *72*, 016502.
[7] a) P. Gilbert, *J. Theor. Biol.* **1972**, *36*, 105-117; b) A. V. Lakshminarayanan, A. Lent, *J. Theor. Biol.* **1979**, *76*, 267-295; c) G. Haberfehlner, A. Orthacker, M. Albu, J. Li, G. Kothleitner, *Nanoscale* **2014**, *6*, 14563-14569.
[8] a) K. J. Batenburg, S. Bals, J. Sijbers, C. Kübel, P. A. Midgley, J. C. Hernandez, U. Kaiser, E. R. Encina, E. A. Coronado, G. Van Tendeloo, *Ultramicroscopy* **2009**, *109*, 730-740; b) A. Zürner, M. Döblinger, V. Cauda, R. Wei, T. Bein, *Ultramicroscopy* **2012**, *115*, 41-49.
[9] K. Thompson, D. Lawrence, D. J. Larson, J. D. Olson, T. F. Kelly, B. Gorman, *Ultramicroscopy* **2007**, *107*, 131-139.
[10] R. Verberne, D. W. Saxey, S. M. Reddy, W. D. A. Rickard, D. Fougerouse, C. Clark, *Microsc. Microanal.* **2019**, *25*, 539-546.
[11] B. W. Krakauer, D. N. Seidman, *Physical Review B* **1993**, *48*, 6724-6727.
[12] M. K. Miller, D. Reinhard, D. J. Larson, *J. Nucl. Mater.* **2015**, *462*, 428-432.
[13] K. Siuzdak, M. Szkoda, A. Lisowska-Oleksiak, K. Grochowska, J. Karczewski, J. Ryl, *Appl. Surf. Sci.* **2015**, *357*, 942-950.
[14] Y.-C. Nah, A. Ghicov, D. Kim, S. Berger, P. Schmuki, *J. Am. Chem. Soc.* **2008**, *130*, 16154-16155.
[15] a) X. Wang, C. Bommier, Z. Jian, Z. Li, R. S. Chandrabose, I. A. Rodríguez-Pérez, P. A. Greaney, X. Ji, *Angew. Chem. Int. Ed.* **2017**, *56*, 2909-2913; b) W. Wu, M. Wang, J. Ma, Y. Cao, Y. Deng, *Adv. Electron. Mater.* **2018**, *4*, 1800185.
[16] a) D.-N. Pei, L. Gong, A.-Y. Zhang, X. Zhang, J.-J. Chen, Y. Mu, H.-Q. Yu, *Nat. Commun.* **2015**, *6*, 8696; b) Y. Nie, L. Li, Z. Wei, *Chem. Soc. Rev.* **2015**, *44*, 2168-2201.

Author Contributions

+J.L. and S.-H. K. contributed equally. J.L. performed the synthesis and the (S)TEM work (HAADF imaging, EELS, EDS, tomography). R. A. A. contributed to the synthesis. S.-H. K. performed the co-electrodeposition, atom probe specimen preparation, and analysis with support from L.S. and B.G., O. K., and P.-P. C. supported on electroplating and co-electrodeposition process. O.K. performed XPS analysis. J.L., S.-H. K., C.S., and B.G. designed overall experiment and drafted the manuscript. All authors then contributed and have given approval to the final version of the manuscript.
